# Biomarkers for precision immunotherapy in the metastatic setting: hope or reality?

**DOI:** 10.3332/ecancer.2020.1150

**Published:** 2020-12-03

**Authors:** Elham Sajjadi, Konstantinos Venetis, Cristian Scatena, Nicola Fusco

**Affiliations:** 1 Divison of Pathology, European Institute of Oncology (IEO) IRCCS, University of Milan, Via Giuseppe Ripamonti 435, 20141 Milan, Italy; 2 Department of Oncology and Hemato-Oncology, University of Milan, Via Festa del Perdono 7, 20122 Milan, Italy; 3 Division of Pathology, Department of Translational Research and New Technologies in Medicine and Surgery, University of Pisa, Via Roma 57, 56126 Pisa, Italy

**Keywords:** biomarkers, immunotherapy, PD-L1, TILs, mismatch repair, TMB

## Abstract

Precision immunotherapy is a crucial approach to improve the efficacy of anti-cancer treatments, particularly in the metastatic setting. In this respect, accurate patient selection takes advantage of the multidimensional integration of patients’ clinical information and tumour-specific biomarkers status. Among these biomarkers, programmed death-ligand 1, tumour-infiltrating lymphocytes, microsatellite instability, mismatch repair and tumour mutational burden have been widely investigated. However, novel tumour-specific biomarkers and testing methods will further improve patients’ outcomes. Here, we discuss the currently available strategies for the implementation of a precision immunotherapy approach in the clinical management of metastatic solid tumours and highlight future perspectives.

## Introduction

Immune checkpoint inhibition has been increasingly applied in several solid tumours, with significant survival benefits, providing a precise patient selection [1–3]. Hence, not all the patients, even in the presence of similar clinical characteristics, would respond in the same way to the same immunotherapy protocol [4]. Furthermore, the toxicity and adverse events of such agents are not uncommon and should be taken into account while assessing the patient’s eligibility [5, 6]. In this scenario, the application of tailored immunotherapy schemes is of great importance.

**Figure 1.**
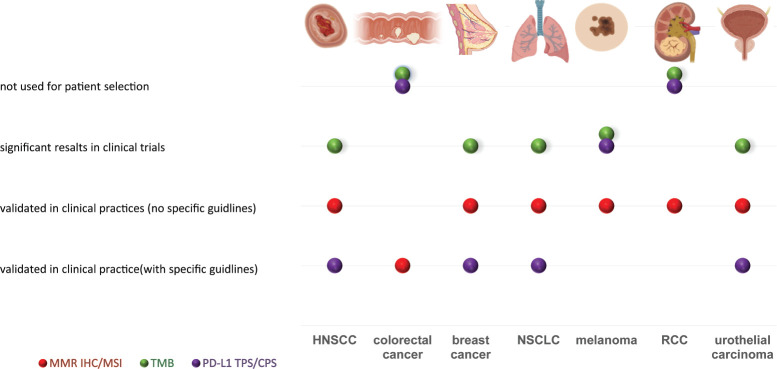
Schematic representation of the main fields of applications of MMR/MSI, PD-L1, TMB testing in patients’ selection for immunotherapy. Tumours are depicted in the columns, while the application of the test in the rows. The colour-coded circles refer to the selected testing method provided on the bottom left legend. The circles are distributed among different anatomical sites based on their clinical utility, as reported on the column placed on the left. MMR, mismatch repair; MSI, microsatellite instability; PD-L1, programmed cell death ligand 1; TMB, tumour mutational burden; IHC, immunohistochemistry; HNSCC, head and neck squamous cell carcinoma; NSCLC, non-small cell lung cancer; RCC, renal cell carcinoma.

In this era of histology-agnostic approvals, the identification of tumour-specific biomarkers and interpretation guidelines is a growing opportunity [7, 8]. Currently, the most studied immune-related biomarkers include programmed death-ligand 1 (PD-L1), tumour-infiltrating lymphocytes (TILs), microsatellite instability (MSI), mismatch repair (MMR) and tumour mutational burden (TMB) [9]. The level of approval of these tests is shown in Figure 1. There are currently multiple lines of evidence on the overall better response rate of TMB-high, MSI-high and PD-L1POS tumours treated with immunotherapy [10, 11]. Additionally, there are several indications that candidate complementary and/ or surrogate biomarkers (e.g. phosphatase and tensin homologue ) may contribute to an optimal patient selection [12–16]. Novel means of mutation measurement as comprehensive genomic profiling (CGP) are currently being explored in this setting [17].

Tumour-specific biomarkers, coupled with companion diagnostics (CDx), may enhance the process of precise patients’ selection, leading to a higher probability of reaching satisfying clinical outcomes [18]. In this review article, we illustrate the impacts and gaps of biomarkers suggested by previous clinical trials and translational research studies in immuno-oncology treatments. A particular focus will be given on the hopes and facts behind the concept of ‘precision immunotherapy’.

## Immunotherapy in clinical practice

Cancer cells can evade the immune system through downregulation or loss of tumour antigens and alterations in the expression of costimulatory and coinhibitory molecules [19, 20]. Under normal conditions, antigens conjugated with major histocompatibility complex (MHC) molecules are presented on the surface of cancer cells. These antigens can be recognised by T cells possessing the same MHC alleles through T-cell receptors–antigen/MHC interactions [21]. For an optimal T cell response, a second signal mediated by co-stimulatory molecules is required. CD28 binds to CD80 and/or CD86, which are present on the surface of activated antigen-presenting cells [22]. Cytotoxic T-lymphocyte antigen 4 (CTLA4) is homologous to CD28 and similarly binds to CD80 or CD86, preventing the attachment of CD28 to these surface proteins. In other words, CTLA4 is a negative regulatory molecule of T cell activation [22, 23]. The pharmacologic inhibition of CTLA4 is one of the possible approaches employed in cancer immunotherapy [22]. The checkpoint axis programmed cell death protein 1 (PD-1)/PD-L1 is another widely explored target [24]. When PD-1 binds to its ligands named as PD-L1 and PD-L2, T cells undergo a negative regulatory process referred to as immune checkpoint [25]. Antibodies that block PD-1 or PD-L1 lead to activation of T cells which can subsequently recognise and attack cancer cells [26]. The therapeutic antibody ipilimumab, targeting CTLA-4, is the first approved checkpoint inhibitor for clinical use in melanoma [27]. Additionally, anti-PD-1 molecules for the management of malignancies such as non-small cell lung cancer (NSCLC), renal cell carcinoma (RCC), Hodgkin lymphoma, melanoma, urothelial carcinoma, metastatic colorectal cancer and hepatocellular carcinoma are Food and Drugs Administration (FDA)-consented to be prescribed [28], as summarised in Table 1. Clinical use of immune checkpoint inhibitors (ICPis) may bring along undesired side effects termed as immune-related adverse events (irAEs) [29]. Reportedly, anti-CTLA-4 therapy often results in more severe side effects comparing to other immunotherapy agents [30]. Organs such as intestine, liver, lung, skin and endocrine glands are frequently affected by immunotherapy toxicity [31]. Around 13%–17% of NSCLC patients treated with anti-PD-1 experienced grade 3 or higher toxicities [32]. Yet, less than 20% of patients show high-grade toxicity when treated with anti-PD-1 and/or anti-PD-L1 [33]. Most of the side effects are tackled by corticosteroids and other adjunctive medications effectively [34].

## The quantum leap of immune-related biomarkers

### Programmed death ligand 1 (PD-L1)

**Figure 2.**
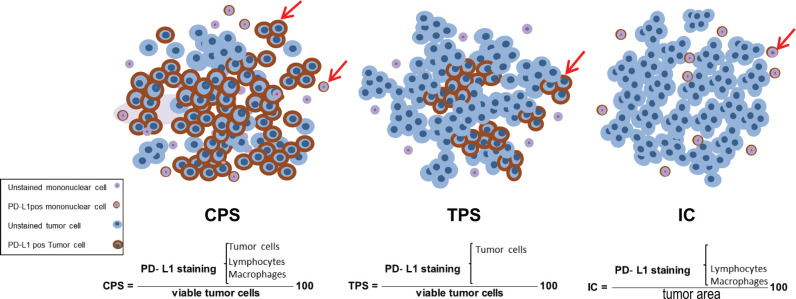
Schematic representation of the available scoring criteria for PD-L1 assessment. CPS counts for both tumour and mononuclear cells which are PD-L1^pos^ among total viable tumour cells, multiplied by 100. While TPS and IC are contributed to PD-L1^pos^, tumour cells and mononuclear cells, respectively, divided by total number of viable tumour cells. PD-L1 stained and unstained tumour and mononuclear cells are depicted on the left bottom legend. PD-L1, programmed cell death ligand 1, CPS, combined positive score; TPS, tumour proportion score; IC, immune cell.

In 2015, FDA approved pembrolizumab as the first PD-1 inhibitor in NSCLC [35]. Since then, different clones of the antibody against PD-1 ligand, such as SP142 (Ventana Medical Systems), SP263 (Ventana Medical Systems) and 22C3 (Dako North America, Inc.) were validated as specific biomarkers for patient selection [36]. Immunohistochemistry (IHC) assessment of PD-L1 is employed for patient selection in several cancers [37]. PD-L1 evaluation differs in each tumour type, thus a conclusive protocol may not fit all malignancies. For instance, tumour proportion score (TPS) which is functional in lung cancer cannot be tailored for head and neck cancer, and *vice versa* for the combined positive score (CPS) [38]. TPS considers PD-L1-positivity merely in neoplastic cells, whereas CPS considers the positivity of tumour cells, lymphocytes and macrophages. CPS equals the number of PD-L1 positive tumour cells and lymphocytes, divided by the total number of viable tumour cells, multiplied by 100. Another example is represented by triple-negative breast cancers (TNBC), where the CDx test for this indication was PD-L1 (SP142) IHC Assay by using the immune cell (IC) scoring system [39]. IC scoring was considered as positive, for those with the presence of PD-L1^POS^ ICs that covered more than 1% of the tumour area (tumour cells, associated intratumoural and contiguous peritumoural stroma) [2]. The PD-L1 scoring systems are shown in Figure 2. Pre-analytical and informative phases of PD-L1 testing have been coordinated in NSCLC where the propagative application of PD-L1 testing in clinical practices indicated coinciding results, mostly by using the 22C3 antibody clone [40]. PD-L1 plays a significant role in the NSCLC treatment profile. In this malignancy, PD-L1 expression is assessed by TPS of membrane expression [41]. Based on KEYNOTE‑042 (NCT02220894), pembrolizumab is approved as the first-line treatment of stage III NSCLC patients with no epidermal growth factor receptor (EGFR) or anaplastic lymphoma kinase (ALK) genomic aberrations, while also the tumour must express PD-L1 (TPS ≥1%) [42].

PD-L1 assessment is debated among scientists. In head and neck squamous cell carcinoma (HNSCC), irrespective of PD-L1 expression status, immunotherapy with nivolumab and pembrolizumab is consented by FDA for the second-line treatment of recurrent and/or metastatic HNSCC [43]. These agents show a greater overall survival (OS) in comparison with the standard, single-agent treatment in those with platinum-refractory, recurrent or metastatic HNSCC [44]. According to Ferris *et al* individuals treated with nivolumab, regardless of tumour PD-L1 expression, appeared to have greater OS than those treated with standard therapy. However, they noted that patients with a tumour PD-L1 expression level of more than 1% may benefit more from nivolumab therapy than those whose PD-L1 level was less than 1% [45].

In HNSCC, CPS is recommended for PD-L1 evaluation, where CPS > 20 represents a significantly longer OS [46]. In melanoma, tumours with PD-L1-overexpression are related to a fairly high response rate (>50%) and longer progression-free survival (PFS) and OS [47]. In PD-L1 positive, advanced and refractory gastric cancer (GC), those treated with pembrolizumab presented a greater objective response rate (ORR). Added to this, PD-L1 negative cases had also shown responses [48]. Regarding RCC, a meta-analysis comprising 4,063 patients suggested a greater OS and PFS in PD-L1 positive tumours [49]. In urothelial carcinoma, patients with high PD-L1 expression had a greater ORR and OS rate [50]. In TNBC, studies indicate that PD-L1 is highly expressed which suggests a potential role for immunotherapy [51]. Pembrolizumab implies durable anti-cancer effects in a small subset of PD-L1 positive metastatic TNBC [52].

### Tumour-infiltrating lymphocytes (TILs)

Leukocytes are thought to be involved in both protumour and antitumour activities [53–55]. Molecular factors formed by ICs may lead to cancer cells’ fate of death or survival [4]. Lymphocytes migrated within tumour stroma or the tumour itself are termed as TILs [56]. In 2014, the International TILs Working Group (ITWG) suggested a standardised methodology for evaluating TILs with detailed information and instruction with step to step tutorial in breast cancer setting [57]. Later on, in 2017, other solid tumours were also included in the ITWG study framework [58] along with other studies confirmations or updates [59, 60]. Accordingly, TILs assessment is performed on haematoxylin and eosin slides by considering both the stromal and the intra-tumour cell compartments [61]. Stromal TILs (sTILs) refer to the area occupied by mononuclear inflammatory cells over the total stromal area, while intra-tumoural TILs (iTILs) are related to the tumour cell area [62]. sTILs and iTILs ought to be reported separately to avoid the effect of tumour cell density and growth pattern on the TIL count. Another reason for reporting individually is that in many tumours the density of TIL is different in both compartments [57]. After defining stromal and intra-tumoural areas with low magnification, the type of inflammatory infiltrates is supposed to be determined [61]. Based on the tumour type, either TILs subtypes or one of them needs to be evaluated. For example, in breast cancer, only sTILs provide valuable information [57]. Apart from TILs, other complementary biomarkers such as the CD4, CD8 and forkhead box P3 are of great relevance in the assessment of TILs function [63, 64].

The lymphocytic infiltration in primary cutaneous melanoma was originally noted by Clark *et al* in almost half a decade ago [65]. Later on, Day *et al* [65] provided data that highlighted the prognostic significance of infiltrated lymphocytes within tumours . The College of American Pathologist has divided TILs in melanoma into three groups, namely Brisk (i.e. diffuse permeation of the invasive tumour), non-Brisk (i.e. focally infiltrating lymphocytes) and not identified subsets [66]. A recent meta-analysis demonstrated non-brisk TILs as a favourable prognostic biomarker in melanoma [64].

In breast cancer, the presence of TILs has been thoroughly investigated, leading to interesting insights. Specifically, increased levels of TILs in TNBC have been associated with better OS and disease-free survival [67]. Another interesting study suggested that sTILs can identify a subset of stage I TNBC patients with exceptional prognosis without adjuvant chemotherapy [68]. Moreover, early-stage HER2^POS^ breast cancer patients with the presence of TILs have been found to benefit when treated with trastuzumab and chemotherapy [69, 70]. However, according to De Angelis *et al* [71] HER2^POS^ breast cancers with the presence of TILs above the threshold of 60%, established by the authors, were marginally associated with higher pathologic complete response rate when treated with lapatinib plus trastuzumab.

In GC, sTILs positivity has been associated with favourable prognosis [72, 73]. According to a systematic review and quantitative meta-analysis, including 43 studies, it has been suggested that high-density TILs also present a favourable prognosis in colorectal cancer [74]. In patients with high-grade serous carcinoma of the ovary, TILs levels may be associated with chemotherapeutic sensitivity [75]. Interestingly, TILs have also been reported as a predictive biomarker of response to anti-PD1 therapy in patients with metastatic NSCLC or metastatic melanoma [76]. However, in RCC, high TILs expression has been suggested to be correlated with poor prognosis [77]. All these studies make evident the extremely important role of TILs across different cancer types while they highlight the need for the discovery of essential information hidden behind TILs evaluation.

Finally, in malignant pleural mesotheliomas (MPMs), low CD4^POS^ and high CD8^POS^ sTILs are associated with poor patients’ survival [78]. In MPMs PD-L1 CPS > 1, stromal CD8^HIGH^ seems to be a poor prognostic factor, while stromal CD4^POS^ peritumoural TILs correlate with a worse prognosis [78]. In these tumours, a high CD4^POS^/CD8^POS^ ratio in the immune microenvironment is an independent prognostic factor for survival. All these recent observations provide novel insights into the clinical scenario of immune-related biomarkers in MPM.

### MMR deficiency and MSI

During the DNA recombination process, strands may detach and reanneal incorrectly, leading to mismatches [79]. However, during evolution, cells have developed strategies to identify and repair these errors. Within this DNA repair network, the mismatch repair (MMR) system is capable of solving insertion/deletion or base-base disparities on DNA [79, 80]. Six MMR proteins—mutL homologue 1 (MLH1), mutL homologue 3 (MLH3), mutS homologue 2 (MSH2), mutS homologue 3 (MSH3), mutS homologue 6 (MSH6) and postmeiotic segregation increased 2 (PMS2)—work coordinately within five complexes to repair mismatches [81]. Deficiency in the compartments of this system may result in modifications in repeated-sequence motifs, termed as microsatellites [79, 80].

Replication errors are more probable in microsatellites due to their repeated structure [82]. Hence, they are considered a potential biomarker for identifying MMR malfunction. The presence of multiple alterations in the length of microsatellites is defined as MSI [83]. MMR/MSI testing is utilised mainly to identify potential Lynch syndrome families. MLH1, MSH2, MSH6 and PMS2 proteins are assessed by IHC antibodies. This evaluation is preferred as one of the most cost-effective and available means of measurement [84]. MSI detection is generally performed through polymerase chain reaction (PCR) approaches by amplifying microsatellite markers with PCR-based methods and detecting MSI by measuring the length of the fragments [85]. Next-generation sequencing (NGS) with higher sensitivity is also being used to detect MSI in various malignancies [86]. In colorectal [87], ovarian [88], endometrial [89] and GC [90], MMR malfunction/MSI is reported as a prognostic biomarker. Contrary, it has been shown that in breast cancer, IHC and MSI testing are not interchangeable tests meaning that each type of cancer requires different and optimised management [8, 91].

The role of gene signature evaluation has become more blatant when FDA related novel immunotherapies to MMR and MSI status regardless of primary tumour site [92]. For the first time in 2017, the FDA approved the use of immunotherapy based on patients’ MMR/MSI status. Accordingly, MMR-deficient and MSI-high metastatic colorectal cancer with progression following treatment with fluoropyrimidine, oxaliplatin and irinotecan were permitted for anti-PD-1 treatment. This accelerated approval was related to nivolumab (OPDIVO, Bristol-Myers Squibb Company) [93]. Later on, in 2018, another accelerated approval was granted, adding ipilimumab (YERVOY, Bristol-Myers Squibb Company Inc.) as a combination therapy to nivolumab of those patients previously noted in 2017 [94] (Table 1).

### Tumour mutational burden (TMB)

The concept of TMB refers to the number of somatic coding DNA mutations in the tumour exome [95]. TMB is noted as a beneficial biomarker in tumour immunotherapy [96]. Genetically unstable characteristics of cancer cells raise the possibility of somatic mutations resulting in neoantigens [97]**.** Diverse types of tumours display a different load of somatic mutations [97]. To date, melanoma and NSCLC show the highest frequencies of mutations [98]. As PD-L1 expression is reported to be highly heterogeneous, predicting the efficacy of immune checkpoint inhibitors (ICPis) in NSCLC is not yet feasible by this biomarker. Hence TMB has shown a new perspective in identifying the most fitting candidates for immunotherapy [99]. According to Hellmann *et al* [100], combination therapy of nivolumab and ipilimumab results in a greater PFS in high TMB cases. Remarkably, this study considered patients regardless of PD-L1 expression . Another study indicated a positive relation between atezolizumab efficacy and high level of TMB, resulting in improved ORR and duration of response in other tumours [101]. These findings suggested the importance of TMB assessment regardless of PD-L1 expression.

Generally, TMB is performed on the DNA extracted from tumour tissue, however, the analysis of circulating tumour DNA (ctDNA) is being investigated in the clinical practice, particularly in follow-up settings [102, 103]. The gold standard method for assessing TMB is whole-exome sequencing (WES) by using NGS technology [104]. This technology estimates the neoantigen load based on somatic nonsynonymous coding mutations [95]. WES highlights the presence of mutations in around 22,000 genes which makes it an expensive and time-consuming application to run [95, 97]. Targeted NGS panels are being used routinely in the clinic for oncogenic mutation detection [97]. A standardised guideline that clearly states methods and analytical validation are of importance as there are several platforms with similar targeted panels and technologies [105].

### CGP assays

CGP is a targeted assay with great value in personalised cancer care transformation [106]. This assay identifies genomic alterations including mutations, copy number variants (amplification) and fusions (rearrangements), associated with targeted therapy opportunities in clinically relevant cancer genes [107]. TMB reports the number of mutations per megabase. However, there is no agreed threshold in existing assays with similar intended use [108]. Friends of Cancer Research and Quality Assurance Initiative Pathology joined to come up with harmonise and standardise TMB testing results [109]. FoundationOne® CDx is an approved CDx test by FDA [110]. This CDx identifies genetic alterations in 324 genes, MSI and TMB by extracting DNA from formalin-fixed paraffin-embedded tumour tissue specimens. The sequenced DNA is then evaluated for the presence or absence of mutations [108]. Another FDA-approved testing panel is IMPACT which utilises NGS to identify the presence of mutations in 468 unique genes, as well as other molecular changes [111]. This assay has more than 99% accuracy with the ability to detect mutations at a frequency of 2 to 5 percent [111]. Rizvi *et al* [112] showed that TMB quantified by targeted NGS closely correlates with TMB as quantified by WES. However, not all NGS panels may be well suited to estimate TMB.

## Biomarkers and precision immunotherapy future prospectives (hope)

ICPis therapies have significantly improved precise treatment in several types of solid tumours [113]. Immunotherapy based on immune checkpoints is being widely expanded in clinical practice by gaining FDA approval in different antibody settings [114]. As listed in Table 1, PD-L1 was approved by the FDA as a biomarker in the line of predicting response to ICPis in several solid tumours [115]. FDA has also approved the application of other biomarkers such as MMR and MSI for colorectal cancer in both monotherapy and combination therapy [116]. Added to these, several other biomarkers and therapies are under the process of accelerated approval which is expected to add more value to ICPis therapy in the near future (Table 1).

Mechanisms associated with ICPIs resistance and predictive biomarkers for ICPis therapy are being actively studied [117]. Immunotherapy efficacy is strictly related to the tumour microenvironment (TME) [118]. Hence, studying components within TME is of interest in forthcoming studies. For instance, myeloid-derived suppressor cells (MDSCs), as a component of TME, are associated with ICPIs inhibition [119]. Reportedly, immunotherapy response can be improved by blocking MDSC activity [120]. Also, a correlation between MDSCs expression and poor OS and PFS is noted [121]. Another perspective issue focuses on stimulating T cell responses in which elevated co-stimulatory molecules result in favourable anti-tumour alterations [121]. For example, inducible T-cell co-stimulator, an indicator of T cell-mediated immune response, that enables early prediction of therapeutic response over multiple treatment regimens [122]. The combination of epigenetic modulator inhibitors with ICPis represents another promising approach in cancer management; as epigenetic alterations may downregulate tumour antigens by disturbing immune recognition [123]. Hong *et al* [124] used nivolumab in order to target epigenetic modulators which significantly increased apoptosis. The application of neoantigen vaccines as modulators of the immune microenvironment is another upcoming topic. Neoantigens resulted in mutations, may give rise to immune responses [125]. As a result, synthesised peptides may induce CD4^POS^ and CD8^POS^ T cell responses [126]. Reportedly, low mutation load and low T cell infiltrating TME are suitable candidates for vaccination [127]. Genetically engineered oncolytic viruses are also of interest. OVs destroy tumour cells by selectively replicating in these cells and inducing systematic anti-tumour immune responses [128]. Several clinical trials are under investigation in combining OV with cancer immunotherapies [129]. Last but not least, gut microbial alterations may lead to the additional possibility of cancer treatment. The gut microbiome is considered as a potential biomarker for ICPis response [121]. Modulation of the gut microbiome to enhance therapeutic response is being tested in multiple ongoing clinical studies [130]. Accordingly, antibiotic consumption before ICPIs had worse OS than unexposed patients [131].

Several studies suggest potential improvement of ICPis efficacy in combination with treatments such as chemotherapy, radiation and targeted therapy. These treatments can modulate the TME resulting in increased immunogenicity [132, 133]. Thus, upcoming findings in novel combinations of therapeutic agents may hopefully unravel the current gap of partial effectiveness of single-agent ICPis therapy [134]. Chemotherapy and radiotherapy are not only able to kill cancer cells directly but also present immunomodulatory properties [135]. Destruction of cancer cells with chemotherapy agents can be followed by the release of tumour-associated antigens that activate immune response as well as reduction of immunosuppressive cells such as MDSCs and Tregs [136, 137]. Radiation not only causes the release of tumour antigens but also improves antigen presentation and TIL infiltration stimulating an immune response [138]. Interestingly, studies have tested the efficacy of either chemotherapy plus ICPis or administration of ICPis after radiotherapy reporting encouraging results [139–141], while high-expectation clinical trials are ongoing (e.g. NCT04262687, NCT03453892). Targeted therapy presents similar immunomodulatory effects [132]. A phase 2 ongoing trial (NCT02954536) evaluated the safety profile and activity of pembrolizumab in combination with trastuzumab and chemotherapy in first-line HER2-positive metastatic gastric, oesophageal and gastroesophageal junction cancer. The response rate of 91% and median OS (27·3 months) were improved compared to the response rate (47%) and median OS (16 months) previously reported for chemotherapy plus trastuzumab. According to this trial, pembrolizumab can be safely combined with trastuzumab and chemotherapy and has promising activity in HER2-positive metastatic esophagogastric cancer [142]. Trastuzumab in combination with pembrolizumab may enhance HER2-specific T-cell responses and improve T cell and dendritic cell trafficking [142]. Other benefits of targeted therapy along with immunotherapy cross-talk could be seen in anti-PD-1 antibody treatment in combination with lenvatinib. This combinatory treatment mainly targets vascular endothelial growth factor and fibroblast growth factor receptors in patients with advanced endometrial cancers. In this study, lenvatinib reduced tumour-associated macrophages and increased the percentage of activated CD8^POS^ T cells secreting interferon [143].

A promising application of ICPis can also be found in neoadjuvant therapy as recent publications note neoadjuvant immunotherapy may result in better clinical efficacy over an adjuvant application ICPis may also be used in the neoadjuvant setting since recent studies support that neoadjuvant immunotherapy can result in better clinical efficacy compared to the corresponding adjuvant therapy [144]. Added to all dated advancements, common means of time-consuming and painful tissue biopsies may be replaced by ctDNA in the peripheral blood [145, 146]. Most tumours are highly heterogeneous and may change during the progression of the disease. To define optimal therapeutic strategies, temporal sampling is mandatory. However, tissue biopsies are not always easy to perform since the tumour site may not be accessible and may not be representative of the whole tumour. Thus, the innovative approach of ‘liquid biopsy’ is gaining more and more attention. The fast turnover of tumour cells leads to a constant release in the peripheral blood of circulating tumour cells (CTCs) and cell-free ctDNA [147]. CTCs are believed to be passively spread from the primary and/or metastatic tumour sites into the bloodstream and may be responsible for the establishment of distant metastases. The liquid biopsy approach allows a repetitive and less invasive interrogation of tumours’ evolution, making sample collection much easier and efficient both for patients and clinicians [148]. All these improvements which are usually based on well-validated principles of certain biomarkers give hope for better results in precision immunotherapy.

## Pitfalls in biomarker-based patients’ selection (reality)

ICPis have drastically transformed cancer treatment profiles by giving hope to physicians in cancer management [149]. However, a significant proportion of patients do not benefit from immunotherapy (with an ORR of only 20% to 23%) [150]. Biomarkers are therefore applied for the finest patient selection. Yet, assortment based on a single biomarker does not appear to be highly efficient [3]. Thus far, numerous gaps should be considered carefully to achieve optimal therapeutic benefit [151]. As stated by Pagni *et al* [4] ‘we do need biomarkers’ to target immune-related pathways in precise therapy. PD-L1 plays a great role as a biomarker [151]. Despite the availability and low cost of PD-L1 assessment by IHC, several technical issues are related to this method. Firstly, the IHC assessment of PD-L1 has limited accuracy due to tumour heterogeneity [151]. Moreover, several antibody clones produced by different companies are used in clinical trials; this variety of antibody clones is mystifying [153]. Added to this, different scoring methodologies—iTILs, sTILs, pTILs—which vary in different tumour types, potentially lead to confusion [154]. Besides, the PD-L1 assessment by itself does not grant to come up with an optimal therapeutic strategy [155].

Resistance to pharmacotherapy is a major issue that prevents a significant subset of patients from responding to PD-1/PD-L1 blockade. Thus, tumour immune microenvironment classification may lighten up the reasons behind [156]. When PD-L1 expression is accompanied by the presence of TILs, it characterises an adaptive resistance of tumours related to the PD-1 pathway (type-I). When both PD-L1 and TILs are not sufficient, termed as immunologically ignorant, ICs do not migrate toward cancer cells (type II). Positive PD-L1 and negative TILs lead to the induction of PD-L1 expression in tumour cells (type III). Contrary, low PD-L1 expression with optimal TILs is referred to as tolerance since the present TILs do not induce PD-L1 expression (type IV) [157]. Ultimately, the goal is to harmonise the patient’s TME with sufficient PD-L1 and TILs [158]. Added to these, not only ICPis response may remain temporarily, with the median duration of response of 1 to 2 years in NSCLC, but it can also result in resistance after the initial response [159]. The mechanisms behind therapeutic resistance are essential to address details of current misfunctions. Yet, introducing proper immunotherapeutic agents and related biomarkers to highlight malfunction is of necessity [160].

Several studies have reported TILs as a potential prognostic and predictive marker in various types of cancer [66]. Even though the TILs working group recommended standardised methodologies for the assessment of immuno-oncology biomarkers/TILs in different malignancies, the efficacy of this evaluation is suggested to be assessed by a large cohort of studies on all solid tumours [161]. MMR-wise, different methods of evaluation such as IHC, MSI and TMB are introduced to evaluate MMR status, hence a single method of assessment could provide more uniform and reliable results [81]. Several institutions perform TMB measurements mostly based on targeted NGS [97]. Despite WES is the gold standard method, usually, it is time-consuming and not affordable to run routinely [104]. Moreover, dedicated platforms are not available in all pathology laboratories [162]. As an alternative, panel-based NGS assays are of use to measure TMB. However, TMB levels are variable among each tumour type and cut-off values need to be established to reliably assess this emerging biomarker. [163]. Regarding adverse events, likewise other medications, ICPis administration brings along unwanted effects [164]. Auto-immune reactions are among the most common side effects and they can be presented as simple skin rashes but also as severe neurologic, hematologic, cardiac and respiratory implications [165]. These can be initiated by nonspecific activations of the immune system through different mechanisms. It is of note that about 2% of irAEs lead to treatment-related deaths, varying by ICPis [33]. Above all, further irAE may have not been documented yet as ICPis have only recently been introduced in therapeutic schemes. Consequently, a more detailed investigation is needed to fully approve ICPis safety profile [149].

The excessive cost of immunotherapy can be considered another important limiting factor [162]. Despite great importance, the economical aspect of this therapy has not been shielded to date [166]. ICPis therapies ought to be bearable so that not only patients can benefit from the latest therapies but also scientists could implement expanded databases for additional validations of their investigations.

## Conclusion

Cancer is a complicated malignancy that involves several mechanisms and immune-related pathways. Therefore, a combination of innovative therapeutic strategies that rely on precise biomarkers has to be developed to profoundly address this issue [167]. Precision immunotherapy has already started to light up a new era in cancer management. It is fair to conclude that several struggles are yet to be addressed in patients’ selection for immunotherapy. We highlight the importance of implementing tumour-specific tests and precise guidelines in routine clinical practice for optimal therapeutic outcomes.

## List of abbreviations

PD-L1, Programmed death-ligand 1; TILs, Tumour-infiltrating lymphocytes; MSI, Microsatellite instability; MMR, Mismatch repair; TMB, Tumour mutational burden; CGP, Comprehensive genomic profiling; MHC, Major histocompatibility complex; CTLA4, Cytotoxic T-lymphocyte antigen 4; PD-1, Checkpoint axis programmed cell death protein 1; NSCLC, Non-small cell lung cancer; RCC, Renal cell carcinoma; FDA, Food and Drugs Administration; irAEs, Immune-related adverse events; ICPis, Immune checkpoint inhibitors; IHC, Immunohistochemistry; TPS, Tumour proportion score; CPS, Combined positive score; TNBC, Triple-negative breast cancers; ICs, Immune cells; HNSCC, Head and neck squamous cell carcinoma; OS, Overall survival; ORR, Objective response rate; ITWG, International TILs Working Group; sTILs, Stromal TILs; iTILs, intra-tumoural TILs; GC, Gastric cancer; MLH1, mutL homologue 1; MLH3, mutL homologue 3; MSH2, mutS homologue 2; MSH3, mutS homologue 3; MSH6, mutS homologue 6; PMS2, postmeiotic segregation increased 2; NGS, Next-generation sequencing; ctDNA, circulating tumour DNA; WES, Whole exome sequencing; CDx, companion diagnostic; TME, Tumour microenvironment; MDSCs, Myeloid-derived suppressor cells; PFS, Progression-free survival; CTCs, Circulating tumour cells

## Conflicts of interest

Nicola Fusco has received honoraria for consulting/advisory role from Merck Sharp & Dohme (MSD), Boehringer Ingelheim and Novartis. These companies had no role in the design of the study, in the collection, analyses or interpretation of data, in the writing of the manuscript and/or in the decision to publish the results. All the other authors declare no conflicts of interest.

## Funding statement

This research did not receive any specific grant from funding agencies in the public, commercial, or not-for-profit sectors.

## Figures and Tables

**Table 1.** Summary of immune checkpoint blockade therapies which have been approved by the FDA for being applied in clinical practices. https://www.fda.gov/

Antibody	Immunotherapy	Trading name	Cancer type	Indications	Date of approval
anti–PD-L1	Durvalumab	**O** IMFINZI, AstraZeneca + etoposide and either carboplatin or cisplatin	Extensive-stage small cell lung cancer (ES-SCLC)	First-line treatment	March 30, 2020
		**O** IMFINZI, AstraZeneca Inc.	Unresectable stage III non-small cell lung cancer (NSCLC)	Disease should not be progressed following concurrent platinum-based chemotherapy and radiation therapy	February 16, 2018
		**O** IMFINZI, AstraZeneca UK Limited)	locally advanced or metastatic urothelial carcinoma	disease progression during or following platinum-containing chemotherapy or within 12 months of neoadjuvant or adjuvant treatment with platinum-containing chemotherapy	May 1, 2017
	Avelumab	**O** BAVENCIO, EMD Serono Inc. + axitinib	Advanced renal cell carcinoma (RCC)	First-line treatment	May 14, 2019
		**O** BAVENCIO, EMD Serono, Inc.	Locally advanced or metastatic urothelial carcinoma	Progressed disease during or following platinum-containing chemotherapy or within 12 months of neoadjuvant or adjuvant platinum-containing chemotherapy	May 9, 2017
		**O** BAVENCIO, EMD Serono, Inc.	Metastatic Merkel cell carcinoma (MCC).	The first FDA-approved product to treat this type of cancer. For 12 years and older	March 23, 2017
	Atezolizumab	**O** TECENTRIQ®, Genentech Inc.	Metastatic non-small cell lung cancer (NSCLC)	first-line treatment adults with high PD-L1 expression (PD-L1 stained ≥ 50% of tumour cells [TC ≥ 50%] or PD-L1 stained tumour-infiltrating immune cells [IC] covering ≥ 10% of the tumour area [IC ≥ 10%]), with no EGFR or ALK genomic tumour aberrations.	May 18, 2020
		**O** TECENTRIQ, Genentech Inc. + paclitaxel protein-bound and carboplatin	Metastatic non-squamous non-small cell lung cancer (NSCLC)	First-line treatment for adults (with no EGFR or ALK genomic tumour aberrations)	December 3, 2019
		**O** TECENTRIQ, Genentech Inc. + carboplatin and etoposide	Extensive-stage small cell lung cancer (ES-SCLC)	First-line treatment for adults	March 18, 2019
		**O** TECENTRIQ, Genentech Inc. + paclitaxel protein-bound	Unresectable locally advanced or metastatic triple-negative breast cancer	PD-L1 (SP142) positive	March 8, 2019
		O TECENTRIQ, Genentech, Inc. + bevacizumab, paclitaxel, and carboplatin	Metastatic non-squamous, non-small cell lung cancer (NSq NSCLC)	First-line treatment of patients with no EGFR or ALK genomic tumour aberrations.	December 6, 2018
		O TECENTRIQ, Genentech Inc.	Locally advanced or metastatic urothelial carcinoma	Not eligible for cisplatin-containing chemotherapy, and whose tumours express PD-L1 (PD-L1 stained tumour-infiltrating immune cells [IC] covering ≥5% of the tumour area) Or Not eligible for any platinum-containing therapy regardless of level of tumour PD-L1 expression	August 16, 2018.
		**O** TECENTRIQ, Genentech Oncology	Metastatic non-small cell lung cancer (NSCLC)	Progressed disease during or following platinum-containing chemotherapy EGFR or ALK genomic tumour aberrations with disease progression	October 18, 2016
		**O** TECENTRIQ, Genentech Inc.	Locally advanced or metastatic urothelial carcinoma	Disease progression during or following platinum-containing chemotherapy or Progression within 12 months of neoadjuvant or adjuvant treatment with platinum-containing chemotherapy	May 18, 2016
anti–PD-1	Nivolumab	**O** OPDIVO, Bristol-Myers Squibb Company.	Metastatic small cell lung cancer (SCLC)	Progression after platinum-based chemotherapy and at least one other line of therapy	August 16, 2018
		**O** OPDIVO, Bristol-Myers Squibb Company	Melanoma	Adjuvant treatment with involvement of lymph nodes or with metastatic disease who have undergone complete resection.	December 20, 2017
		**O** OPDIVO, Bristol-Myers Squibb Co.	Hepatocellular carcinoma (HCC)	Previously treated with sorafenib.	September 22, 2017
		**O** OPDIVO, Bristol-Myers Squibb Co.	Metastatic colorectal cancer	-12 years and older -Mismatch repair deficient (dMMR) and microsatellite instability high (MSI-H) -Malignancy progressed following treatment with a fluoropyrimidine, oxaliplatin, and irinotecan	August 1, 2017
		**O** OPDIVO, Bristol-Myers Squibb Co.	Locally advanced or metastatic urothelial carcinoma	Progression during or following platinum-containing chemotherapy or Have disease progression within 12 months of neoadjuvant or adjuvant treatment with a platinum-containing chemotherapy	February 2, 2017
		O OPDIVO, Bristol-Myers Squibb Co.	Recurrent or metastatic squamous cell carcinoma of the head and neck (SCCHN)	Progression on or after a platinum-based therapy	November 10, 2016
		O OPDIVO, Bristol-Myers Squibb Co	Advanced renal cell carcinoma	Patients who have received prior anti-angiogenic therapy	November 23, 2015
		**O** OPDIVO, Bristol-Myers Squibb Co	Metastatic non-small cell lung cancer (NSCLC)	Progression on or after platinum-based chemotherapy. EGFR or ALK genomic tumour aberrations should have disease progression on FDA-approved therapy for these aberrations prior to therapy	October 9, 2015
		**O** OPDIVO, Bristol-Myers Squibb Co	Unresectable or metastatic melanoma	Progression following ipilimumab and, if BRAF V600 mutation positive, a BRAF inhibitor	December 22, 2014
	pembrolizumab	**O** KEYTRUDA, Merck & Co. Inc	New dosing regimen	400 mg every 6 weeks for pembrolizumab across all currently approved adult indications, in addition to the current 200 mg every three weeks dosing regimen.	April 28, 2020
		**O** KEYTRUDA, Merck & Co. Inc.	Bacillus Calmette-Guerin (BCG)-unresponsive, high-risk, non-muscle invasive bladder cancer (NMIBC)	With carcinoma in situ (CIS) with or without papillary tumours who are ineligible for or have elected not to undergo cystectomy.	January 8, 2020
		**O** KEYTRUDA, Merck + lenvatinib (LENVIMA, Eisai)	Advanced endometrial carcinoma	That is not microsatellite instability high (MSI-H) or mismatch repair deficient (dMMR) Have disease progression following prior systemic therapy but are not candidates for curative surgery or radiation.	September 17, 2019
		**O** KEYTRUDA, Merck & Co. Inc.	Advanced esophageal squamous cell cancer	Tumour PD-L1 expression (Combined Positive Score [CPS] ≥10), determined by an FDA-approved test Disease progression after one or more prior lines of systemic therapy.	July 30, 2019
		**O** KEYTRUDA, Merck & Co. Inc.	Metastatic small cell lung cancer (SCLC)	Disease progression on or after platinum-based chemotherapy and at least one other prior line of therapy.	June 17, 2019
		**O** KEYTRUDA, Merck & Co. Inc.	Metastatic small cell lung cancer (SCLC)	Disease progression on or after platinum-based chemotherapy and at least one other prior line of therapy.	June 17,2019
		**O** KEYTRUDA, Merck & Co. Inc.	Metastatic or unresectable recurrent head and neck squamous cell carcinoma (HNSCC)	First-line treatment	June 10, 2019
		**O** KEYTRUDA, Merck & Co. Inc. + axitinib	Advanced renal cell carcinoma (RCC)	First-line treatment	April 19, 2019
		O KEYTRUDA, Merck & Co. Inc.	Stage III non-small cell lung cancer (NSCLC)	First-line treatment -Not candidates for surgical resection or definitive chemoradiation or metastatic NSCLC. -Patients’ tumours must have no EGFR or ALK genomic aberrations and express PD-L1 (Tumour Proportion Score [TPS] ≥1%) determined by an FDA-approved test.	April 11, 2019
		O KEYTRUDA, Merck & Co. Inc.	Melanoma	Adjuvant treatment -With involvement of lymph node(s) following complete resection.	February 15, 2019
		**O** KEYTRUDA, Merck & Co. Inc.	Recurrent locally advanced or metastatic Merkel cell carcinoma (MCC)	Adult and pediatric patients	December 19, 2018
		**O** KEYTRUDA, Merck & Co. Inc.	Hepatocellular carcinoma (HCC)	Previously treated with sorafenib	November 9, 2018
		**O** KEYTRUDA, Merck & Co. Inc. + carboplatin and either paclitaxel or nab-paclitaxel	Metastatic squamous non-small cell lung cancer (NSCLC)	First-line treatment	October 30, 2018
		**O** KEYTRUDA, Merck & Co. Inc. + Pemetrexed,platinum	Metastatic, non-squamous non-small cell lung cancer (NSqNSCLC)	First-line treatment with no EGFR or ALK genomic tumour aberrations	August 20, 2018
		**O** KEYTRUDA, Merck & Co. Inc	Locally advanced or metastatic urothelial cancer	PD-L1 levels evaluation in tumour tissue who are cisplatin-ineligible. PD-L1 expression CPS ≥ 10 as determined by an FDA-approved test Or not eligible for any platinum-containing chemotherapy regardless of PD-L1 status	August 16, 2018
		**O** KEYTRUDA, Merck & Co. Inc	Refractory primary mediastinal large B-cell lymphoma (PMBCL)	Treatment of adult and pediatric patients, relapsed after two or more prior lines of therapy.	June 13, 2018
		**O** KEYTRUDA, Merck & Co. Inc	Recurrent or metastatic cervical cancer	Disease progression on or after chemotherapy PD-L1 expression (CPS ≥1) as determined by an FDA-approved test	June 12, 2018
		**O** KEYTRUDA, Merck & Co. Inc	Recurrent locally advanced or metastatic, gastric or gastroesophageal junction adenocarcinoma	PD-L1 expression as determined by an FDA-approved test Disease progression on or after two or more prior systemic fluoropyrimidine- and platinum-containing chemotherapy and, HER2/neu-targeted therapy	September 22, 2017
		O KEYTRUDA, Merck & Co. Inc	Unresectable or metastatic colorectal cancer	Adult and pediatric patients unresectable or metastatic, MSI-H or dMMR solid tumours progressed following prior treatment with no satisfactory alternative treatment options or MSI-H or dMMR colorectal cancer that has progressed following treatment with a fluoropyrimidine, oxaliplatin, and irinotecan.	May 23, 2017
		O KEYTRUDA, Merck & Co. Inc	Locally advanced or metastatic urothelial carcinoma	Disease progression during or following platinum-containing chemotherapy or within 12 months of neoadjuvant or adjuvant treatment with platinum-containing chemotherapy.	May 18, 2017
		**O** KEYTRUDA, Merck & Co. Inc + pemetrexed and carboplatin	Metastatic non-squamous non-small cell lung cancer (NSCLC)	Previously untreated	May 10, 2017
		**O** KEYTRUDA, Merck & Co. Inc	Metastatic non-small cell lung cancer (NSCLC)	Tumours express PD-L1 as determined by an FDA-approved test	October 24, 2016
		**O** KEYTRUDA, Merck & Co. Inc	Recurrent or metastatic head and neck squamous cell carcinoma (HNSCC)	Disease progression on or after platinum-containing chemotherapy	August 5, 2016
		**O** KEYTRUDA, Merck & Co. Inc	Unresectable or metastatic melanoma	.	December 18, 2015
		**O** KEYTRUDA, Merck & Co. Inc	Metastatic non-small cell lung cancer (NSCLC)	Tumours express PD-L1 as determined by an FDA-approved test, with disease progression on or after platinum-containing chemotherapy	October 2, 2015
		**O** KEYTRUDA, Merck & Co. Inc	Unresectable or metastatic melanoma	Disease progression following ipilimumab BRAF V600 mutation positive	September 4, 2014
anti-CTLA4	Ipilimumab	**O** YERVOY, Bristol-Myers Squibb Company	Cutaneous melanoma	Additional indication of adjuvant treatment of patients Pathologic involvement of regional lymph nodes of more than 1 mm who have undergone complete resection, including total lymphadenectomy	October 28, 2015
		**O** YERVOY, Bristol-Myers Squibb Company	Unresectable or metastatic melanoma		March 25, 2011
	Nivolumab + ipilimumab	**O** nivolumab (OPDIVO, Bristol-Myers Squibb Co.) + ipilimumab (YERVOY, Bristol-Myers Squibb Co.) + 2 cycles of platinum-doublet chemotherapy	Metastatic or recurrent non-small cell lung cancer (NSCLC), with no epidermal	As first-line treatment With growth factor receptor (EGFR) or anaplastic lymphoma kinase(ALK) genomic tumour aberrations.	May 26, 2020
Combination therapy		O nivolumab (OPDIVO, Bristol-Myers Squibb Co.) + ipilimumab (YERVOY, Bristol-Myers Squibb Co.)	Metastatic non-small cell lung cancer	As first-line treatment Tumours express PD-L1(≥1%), as determined by an FDA-approved test With no epidermal growth factor receptor (EGFR) or anaplastic lymphoma kinase (ALK) genomic tumour aberrations	May 15, 2020
		O nivolumab (OPDIVO, Bristol-Myers Squibb Co.) + ipilimumab (YERVOY, Bristol-Myers Squibb Co.)	Hepatocellular carcinoma (HCC)	Previously treated with sorafenib..	March 10, 2020
		**O** nivolumab (OPDIVO, Bristol-Myers Squibb Co.) + ipilimumab (YERVOY, Bristol-Myers Squibb Co.)	Advanced renal cell carcinoma	Intermediate or poor risk previously untreated	April 16, 2018
		**O** nivolumab (OPDIVO, Bristol-Myers Squibb Co.) + ipilimumab (YERVOY, Bristol-Myers Squibb Co.)	Unresectable or metastatic melanoma	With BRAF V600 wild-type	September 30, 2015

https://www.fda.gov/

**O** approved

**O** accelerated approva

**O** updated prescribing information

ALK: anaplastic lymphoma kinase; EGFR: epidermal growth factor receptor; HER2: human epidermal growth factor receptor 2
